# On the Nature and Causes of Dysentery

**Published:** 1799-07

**Authors:** J. Christie

**Affiliations:** 27th Foot.


### *On the Nature and Causes of Dysentery: By Mr. J.* CHRISTIE, *(27th Foot.)*

[Concluded from our last Number, pp. 347—356.]

WITH respect to those contagions producing fever, the plague, dysentery, &c. I question whether they be specifically distinct, or whether, from a higher degree of virulence of the same poison, different forms of disease appear.—Thus I have frequently observed, particularly in India, those soldiers who were much addicted to drinking toddy, and other liquors that affect especially the alimentary canal, subject to fluxes; and other soldiers, diers, more temperate, but in other respects exposed to the same exciting causes, attacked with fever, &c. The heat of the atmosphere, when carried to a certain length, certainly has the power of modifying or rendering altogether inert the action of contagious effluvium ; hence within the Tropics, the plague and jail-fever are either unknown, or rendered much less formidable than they would have been in more temperate climates. As I before mentioned, I could never observe the dysenteries in India communicated from one person to another.

As the syphilitic poison is a native of a warm climate, where the passions are readily excited, and generally keen, may it not be probable, that this disease was first produced by excessive and licentious sexual intercourse; by this intercourse being indulged when the habitual changes of the female, and the more acrid secretions of the warm climate, and want of cleanliness, would render the formation of a new poison more likely to happen ? This will be corroborated, by observing how other morbid poisons have been generated by the bodies of men placed under certain circumstances; and this leads me to conjecture that the syphilitic, like other morbid poisons, may also exist only in a gaseous or invisible form ; that its activity is only preserved when diffused in a proper vehicle, as for instance, the serum of our blood ; that this poison is either of so tenacious and sluggish a nature, that the atmosphere has not the power of suspending it in an active state, or what would appear still more probable, there may be a neutralizing quality in the air which has the power of rendering the syphilitic poison inert, the moment it begins to evaporate. The atmosphere possesses, perhaps, many such benign powers, with which we may never become acquainted, and here it would appear at least consonant to the benevolence and wisdom of the Creator of the Universe, to suppose there may be a quality in the air to prevent the ravages of a poison, which, were it allowed to float about actively, would render the pure and virtuous its victim, as well as the revelling debauchee.—While we thus suppose that some of the fluids of our bodies are the only proper vehicle in which certain poisons can exist, we may also be allowed to conjecture, that the air is the only fluid in which others can preserve their peculiar properties, and that, when they come in contact with the fluids of our body, *they* have the power of rendering such poisons inert; and this act of the constitution may produce various kinds of fever, among which we would class intermittents, scarlet fever, hooping-cough, and probably also measles.

Having thus mentioned so fully the appearances of dysentery, and offered those conjectures with regard to its nature and origin, and contagion in general, we now proceed to mention the method of cure that was employed principally at Bombay.

When

When along with other ſymptoms of the diſeaſe, there was foul tongue, nauſea or vomiting, we generally began by giving an antimonial emetic, as a ſolution of four or five grains of the antimonium tartariſatum, diſſolved in four ounces of water; and the patient directed to take an ounce of this every fifteen minutes till he vomited; and if, (as generally happened) it alſo operated by ſtool, ſo much the better.

Commonly, however, on the firſt appearance of the diſeaſe, we began by giving an ounce or an ounce and an half of Glauber's ſalts (natron vitriolatum ph. Lond.) with or without a little manna; and, if the griping continued, ſix drachms or an ounce more of the ſame ſalts was given, to carry off the complaint entirely, although at the firſt attack, there was every appearance of a violent diſeaſe. In all the milder caſes theſe ſalts rarely failed in removing the ſymptoms; and I have long thought the neutral ſalts are peculiarly well adapted for the cure of this diſeaſe. I have therefore been generally in the habit of giving either the Glauber's ſalt or ſal cathartieus amarus, (magneſia vitriolata) in the beginning of dyſenteric affections, and have conſtantly found them preferable to any other purge whatever.

In the dyſenteries I have had occaſion to ſee, in camps in England and on the Continent of Europe, I found that after a purge or two of the neutral ſalts, the giving a weak ſolution of the tartariſed antimony, (as one grain in two ounces of water) in the quantity of an ounce three or four times a day, ſo as to operate chiefly on the bowels, appeared to be well adapted for the cure of dyſentery—ſeldom was any thing elſe neceſſary—it rarely, in ſuch doſes, produced nauſea—it kept the bowels pretty eaſy, and generally occaſioned a ſalutary moiſture on the ſkin, which always mitigates the violence of the dyſenteric ſymptoms.

In thoſe caſes at Bombay, however, when the ſymptoms continued unabated after the purges, we began immediately the uſe of the mercury internally, as well as externally applied. The freedom with which practitioners uſe mercury, in this and other endemics of India, is well known: the generality of its application, and the character it has long held among, perhaps, ſome of the moſt eminent practitioners of the Britiſh empire, ſeem to render it altogether unneceſſary for me to expatiate on the utility of this medicine in dyſentery. On my arrival at Bombay, I was not indeed acquainted with the general uſe of mercury, in the cure of this complaint; and I was therefore employed at firſt in adminiſtering the uſual remedies only, until Doctor Kerr, of H. M. 75th Regiment, a gentleman of great diſcernment and much experience in tropical diſeaſes, cleared up my mind reſpecting its uſe—his directions were regularly followed, and I muſt confeſs with conſiderable heart-felt ſatisfaction to myſelf.

In ſome, where there was more than uſual vaſcular excitement, and where the habit was full and plethoric, we ſometimes took away a few ounces of blood from the arm, and ſeemingly with benefit. In no complaint, however, ſhould the lancet be uſed with greater caution perhaps than in this diſeaſe, for it is apt to be followed by great debilty, and inflammatory ſymptoms ſeldom run ſo high as to urge its uſe.

Immediately then after the purge of the ſalts, (to which was occaſionally added, with advantage, half a grain or ſo of the tartarized antimony) a drachm and a half of the common mercurial ointment was rubbed in morning and evening, at the ſame time giving a pill, conſiſting of two or three grains of calomel, every night at bed time, till the mouth became affected; and in very few inſtances indeed without an immediate relief from all the ſymtoms of the flux.

When however the griping and teneſmus continued violent after the mouth became affected, the mercurial frictions were increaſed and continued till a copious ſalivation was brought on, and always with manifeſt advantage.

When the ſkin felt very hot and parched, together with the calomel pill was added about the third of a grain of the tartar emetic, or what ſeemed to anſwer ſtill better, ſix or eight grains of James's powder. Theſe generally, by introducing an agreeable diaphoreſis, afforded conſiderable relief; beſides, antimonial medicines ſeemed to haſten the action of the mercury, an event always to be wiſhed and looked for, and which, as before mentioned, ſeldom failed in procuring a remiſſion of the dyſenteric ſymptoms;—when theſe however were unuſually violent, and the mercury long and tardy in operating, the calomel pill was given morning and evening, and the mercurial frictions were increaſed to two, or even three drachms of the ſtrong ointment two or three times a day; and, though this was certainly a great quantity of a very powerful remedy, in a very weakening diſeaſe, I never had any cauſe to regret ſuch exhibition, for it frequently ſeemed to act like a charm; and even where there is more than uſual vaſcular excitement, the mercury ſhould never be long withheld or ſcantily exhibited.

When a ſufficient quantity of the mercury could not be rubbed into the legs and thighs, the frictions were then directed to be made on the ſides and abdomen alſo, thus covering, as it were, the whole body with mercurial ointment.—In this way two or three ounces of the ſtrong ointment would be uſed in the courſe of a few days, at the ſame time the calomel pill morning and evening; and, although the effects produced by the medicine, as profuſe ſalivation, hemorrhagy from mouth and throat, and a total incapability of ſwallowing harſh ſolids, theſe were matters of ſecondary conſideration when compared to the relief obtained from the diſtreſſing griping and teneſmus.

In

In some obstinate cases, especially those of long standing, or which had undergone relapses, the mercury could never be made to affect the mouth sufficiently—it merely produced a brassy kind of taste, without an increased discharge of saliva, or moisture on the skin. In some such cases, small doses of James's powder, or the warm bath continued for a sufficient length of time, appeared to promote the action of the mercury, and to relieve the complaint.—Along with the free use of the mercury, we did not fail at the same time to employ other remedies in use in this complaint—The patients drank largely of bland mucilaginous drinks, as barley, or rice water, linseed tea, solution of gum arabic in toast and water, &c.—These, and particularly the oily demulcents, as they have been called, are particularly useful in sheathing the intestines from the acrimony of the matter.—It is seldom however, that the stomach can bear the oily medicines in such quantity as to be of any great service—and the acidulated drinks, as decoction of prunes or tamarinds, so frequently, and, from the general putrid tendency of the disease, so rationally recommended, will seldom be found at all admissible: though at first eagerly desired by the patient, he soon finds such drinks increase the griping intolerably, and he prefers the plainest, which in most cases are the best.

The patient's diet consisted of vegetable food and soups only, as barley, or chicken broth, wheat or rice pudding, sago, and, during the violence of the disease, a total abstinence from animal food.

A large emollient glyster, made of warm water and oil, or a starch glyster, or one made of decoction of linseed with oil, was thrown up the rectum at bed-time. When the griping, stranguary, or tenesmus was very severe, these were repeated two or three times in the course of the day; and, by adding a couple of grains of opium, or about sixty drops of the tincture, to the one given at bed-time, generally procured the patient a refreshing sleep and an easy night.

In some cases, where the frequent stools and severe griping and tenesmus were uncommonly distressing, so as to exhaust the patient in an alarming manner, I was tempted to give an opiate by the mouth; and, although it always procured considerable relief, which the patient was always very sensible of, and of course became eager for, I never gave opium by the mouth in this disease but with reluctance. I always thought it disposed the bowels, after its action, to more severe griping and less free stools. In some cases, however, it was really indispensibly necessary, and in convalescents, where there is chiefly debility of the intestines, with little griping and free stools, opium will be found on many accounts preferable to any other medicine.

When the pain in the bowels is very distressing, this will be generally relieved by warm fomentations, or the warm bath frequently repeated and continued continued for a fufficient length of time. When thefe however fail, relief will be pretty certainly obtained, from the application of a large blifter to the umbilical region—and the good effects of this remedy appeared to me often remarkable, and when the griping returned violent after the firft bliftering, a fecond or third was at the interval of fome days, occafionally repeated, and always with very ftriking benefit.

To this fubject I was led to pay fome attention from having feen in notes, added to firft lines that well deferve to have been written in letters of gold—the good effects of bliftering in griping doubted—and that the advantages faid to arife from this might probably be obtained from the purges and diluents. The effects of a large blifter however, are in general more evident and decifive than that of diluents or purges, and I fee indeed but little room for deception here.—From many and repeated trials I have been long perfuaded, that bliftering, particularly when repeated, has procured moft evident alleviation of the fevere griping, when fuch relief could not be obtained by any other means; and from analogy it appears to me, to be a very rational remedy, by its taking off in fome meafure the inflammatory difpofition of the internal parts, and may alfo be ufeful here perhaps by favouring the more ready abforption of the mercury.

Generally, after the ufe of mercury and other remedies mentioned, the patient gradually recovered without other medicines. In other cafes however the griping, frequent ftools and tenefmus, continued for weeks; and all that was done in fuch cafes, was to keep the patient's bowels pretty eafy by mild laxatives and occafional glyfters in which fome opium was diffolved.—A very ufeful medicine, where the ftomach will bear it, is caftor oil. We were however in fuch cafes generally in the habit of giving a dofe of pills, confifting of three or four grains of calomel with fix or feven of rhubarb every fecond or third day, as occafion required. It fometimes happened that the calomel given in this manner did not operate fufficiently; or, where the patient was confiderably debilitated, it even appeared to act too roughly, and then nothing feemed to anfwer fo well as a gentle dofe of the purging neutral falts; thefe always procured eafy ftools, and left the patient in an eafier ftate than any other laxative.

Rhubarb alone always appeared to me an improper remedy in this difeafe; perhaps it fhould not be given at all, and I could never obferve any advantage to be derived from ipecacuana.

When the bowels continue long in a relaxed ftate, and the patient does not recover his ftrength readily, fome aftringents or tonics may become neceffary; but the mildeft are certainly the beft. The bark, particularly the the Anguftura, the Quaffia, or Simarauba, may be tried; but gradually bringing the patient to a nourifhing diet with a little wine, will in general fuperfede the ufe of other tonics. Indeed it was only in the more violent cafes, or where the mercury was fluggifh in its operation, that other remedies were thought requifite. Thofe were confidered merely as palliatives in the complaint, or auxiliaries to the grand remedy, mercury; the effect of which, in dyfentery, is not a little remarkable. Its modus operandi in this and other difeafes, where there is great debility, and where we might fuppofe the fymptoms would be thereby increafed and aggravated, may be difficult to account for. The quantity diffufed at any time in the fluids, is probably too fmall to have any fhare in altering their chemical properties; and, by faying that mercury, in the cure of difeafes, excites a peculiar action in the fyftem, incompatible with the difeafed one, is going as far perhaps as we are authorifed at prefent. Its influence in fyphilis, in chronic rheumatifm, in yellow fever, and in dyfentery, feem at leaft to juftify the expreffion.

I fhall now conclude thefe obfervations with a few words on the diffection of thofe I have had occafion to examine after death at Bombay. Six bodies were opened, and in all the colon and rectum appeared to be the principal feat of the difeafe. The firft cafe was a man who had been ill two months previous to our landing. The internal furface of the great gut had an irregularly rugous appearance, and was confiderably increafed in thicknefs. The mefenteric glands were indurated and enlarged; and a cyft, containing a confiderable quantity of purulent matter, was found in the right lobe of the liver. Here it may be proper to fay, that this was the only cafe that was examined, where the difeafe had been of long ftanding, all the others having died in the fecond or third week of the difeafe, or at leaft from the time they had relapfes. To fave repetitions, the following were the general appearances:

The internal furface of the colon and rectum was covered with irregular ulcers, of the fize of crown and half-crown pieces. In fome, this inteftine appeared as if the villous coat had been peeled off; and its fubftance was at the fame time much increafed in weight and fize. The external furface of the great inteftine had generally interfperfed over it gangrenous-looking fpots; had loft its texture; was flaccid to the touch. In fome the gut was completely mortified, and in one, the feculent matter had efcaped into the cavity of the abdomen. In this cafe the mefenteric glands were very confiderably enlarged; the liver was indurated, and had little abfceffes formed throughout its fubftance; and the very offenfive putrid fmell arifing from the opening of this body, was intolerable. In fome too, the fmall guts had not not efcaped, for portions of the hium appeared not merely to have been altered to a darker colour, and thickened in their fubftance, but alfo the internal furface partially affected with ulcerations.

In one fubject a confiderable quantity of bloody ferum had been poured out into the cavity of the abdomen.

